# The association of genetically controlled CpG methylation (cg158269415) of protein tyrosine phosphatase, receptor type N2 (*PTPRN2*) with childhood obesity

**DOI:** 10.1038/s41598-019-40486-w

**Published:** 2019-03-19

**Authors:** Suman Lee

**Affiliations:** Center for Genome Science, National Institute of Health, Chungcheongbuk-do, 363-951 Republic of Korea

## Abstract

Protein tyrosine phosphatase, receptor type N2 (*PTPRN2*) encodes a major islet autoantigen in type-1 diabetes. Previous genetic studies have shown its significant association with obesity. PTPRN2 plays an important role in epigenetic regulation of metabolic diseases and cancers. We investigated CpG methylations (cg17429772 and cg158269415) in *PTPRN2* by pyrosequencing from blood samples of childhood obesity (n = 638). cg158269415 had significant positive correlations with body mass index (BMI) and waist-hip ratio (WHR). Case-control analysis showed that cg158269415 methylation in blood sample was significantly more hypermethylated in obese cases (n = 252), an average of 2.93% more than that that in controls (n = 386). The cg158269415 methylation has a trimodal distribution pattern with strong dependency on nearby located rs1670344 G > A genotype. Correlations of cg158269415 with BMI and WHR were significant and strong in major G allele carriers (GG + GA). Our study showed that an epigenetic association of *PTPRN2* gene with childhood obesity was under certain genetic background. The genetic and epigenetic interplay of *PTPRN2* gene may implicate a mechanism of childhood obesity. Whether these small changes in DNA methylation from whole blood are causally or consequently related to childhood obesity outcome and their clinical relevance remains to be determined. However, this study presents a promising obesity risk marker that warrants further investigation.

## Introduction

Obesity is a prevalent health problem affecting modern societies. It is particularly severe for children in developed countries. Overweight children are more likely to have risk factors of chronic diseases including cardiovascular disease and type 2 diabetes later in life than other children^1^.

Protein Tyrosine phosphatase, receptor type N2 (*PTPRN2*) encodes a major islet autoantigen in type-1 diabetes. It is localized to the membrane of insulin-containing dense-core vesicles^2,3^. PTPRN2 plays an important role in insulin secretion in response to glucose stimuli. Its specific substrates and binding partners suggest a variety of its function for metabolism including obesity and type 2 diabetes^4,5^.

Previous genetic linkage study of body mass index (BMI) has shown strong signals from 7q36.3. Sixty-five independent loci associated with obesity have been found (with q < 0.05) by targeted resequencing of chromosome 7q36.3 region in Northern Han Chinese, and twenty-two of them, which located at introns of *PTPRN2*, have significant associations with obesity^6^. Novel candidate loci including *PTPRN2* associated with pediatric obesity have been identified by genome-wide copy number variation (CNV) analysis^7^. By investigating rare (<1%) CNV, *PTPRN2* has been found to be a biologically relevant candidate gene for pediatric obesity.

PTPRN2 gene is epigenetically regulated in various biological processes including tumor pathogenesis^8–12^. Chen *et al*. have reported that different gene expression and DNA methylation of *PTPRN2* in the human placenta are related to the intrauterine environment^8^. Prenatal growth patterns and birthweight are significantly associated with the epigenetic signature of *PTPRN2* in human placentas. *PTPRN2* is up-regulated in highly metastatic breast cancer cells^9^. It can enzymatically change activity in metastatic breast cancer cells and enhance cellular migration and metastatic capacity. Gentilini *et al*. have found that *PTPRN2* is potentially involved in hepatocellular carcinoma pathogenesis by investigating genome-wide DNA methylation profile for 69 pairs of hepatocellular carcinoma cancer and adjacent non-cancerous liver tissues^10^. Hypomethylated *PTPRN2* is significantly over-expressed in tumor tissue compared to that in normal liver tissue^11^. In naive CD4+ T cell, *PTPRN2* cis-acting genetic variants account for some ethnicity-specific variability in DNA methylation^12^. PTPRN2 is hypomethylated in African-American lupus patients compared to European-American decent.

The objective of this study was to investigate epigenetic variations of the *PTPRN2* gene by using target pyrosequencing of up to 638 Korean childhood obesity subjects. We examined obesity-related differentially methylated CpG site (cg158269415) by pyrosequencing to investigate their associations with obesity related traits. The potential role of epigenetic mechanism to individual risk of childhood obesity was explored and background genetic contribution for obesity specific CpG methylation was also determined. Our results revealed a crosstalk between genetic and epigenetic events of *PTPRN2* gene in childhood obesity.

## Experimental Procedures

All methods are approved by the committee of Korea National Institute of Health (KNIH) and carried out in accordance with relevant guidelines and regulations of KNIH.

### Study subjects

Subjects of this study were selected from the Korean Child-Adolescent Cohort Study^13–15^. The overall objective of the cohort study was to identify early risk factors for obesity and associated metabolic diseases in Korean children. Obesity is defined as a BMI at or above the 95th percentile for children and teens of the same age and sex according to the recently proposed definition by the Centers for Disease Control and Prevention. Informed consent was obtained from children’s parents. The Institutional Review Board (IRB) of the Korea National Institute of Health approved this study (IRB approval number: 2014-08EXP-05-P-A). Detailed subject characteristics are presented in Table 1.

**Table 1:** Characteristics of subjects with childhood obesity.

Variables	Controls	Cases	*P*
Age	13.93 ± 0.77	13.94 ± 0.84	0.6805
N (M/F)	386 (192/194)	252 (138/114)	
BMI	20.23 ± 2.58	32.69 ± 3.60	**<0**.**0001**
WHR	0.77 ± 0.05	0.90 ± 0.06	**<0**.**0001**
FPG	92.79 ± 7.03	94.93 ± 12.58	**<0**.**0001**
AST	19.11 ± 3.98	27.10 ± 16.30	**<0**.**0001**
ALT	12.85 ± 11.27	35.59 ± 33.94	**<0**.**0001**
TC	158.05 ± 25.62	175.06 ± 28.29	**<0**.**0001**
TG	73.14 ± 37.96	122.87 ± 65.98	**<0**.**0001**
HDL	56.25 ± 10.25	46.12 ± 8.19	**<0**.**0001**
LDL	87.17 ± 21.98	104.48 ± 24.95	**<0**.**0001**

N(M/F): Number(Male/Female); BMI (kg/m^2^): Body Mass Index; WHR: Waist to Hip Ratio; FPG (mg/dL): Fasting Plasma Glucose; AST(IU/L): Aspartate transaminase; ALT(IU/L): alanine aminotransferase; TC (mg/dL): Total Cholesterol; TG (mg/dL): Triglyceride; HDL (mg/dL): High-density lipoproteins-Cholesterol. Raw P values < 0.05 are shown in bold.

### Quantitation of CpG DNA methylation and genotyping by pyrosequencing analyses

**Figure 1:**
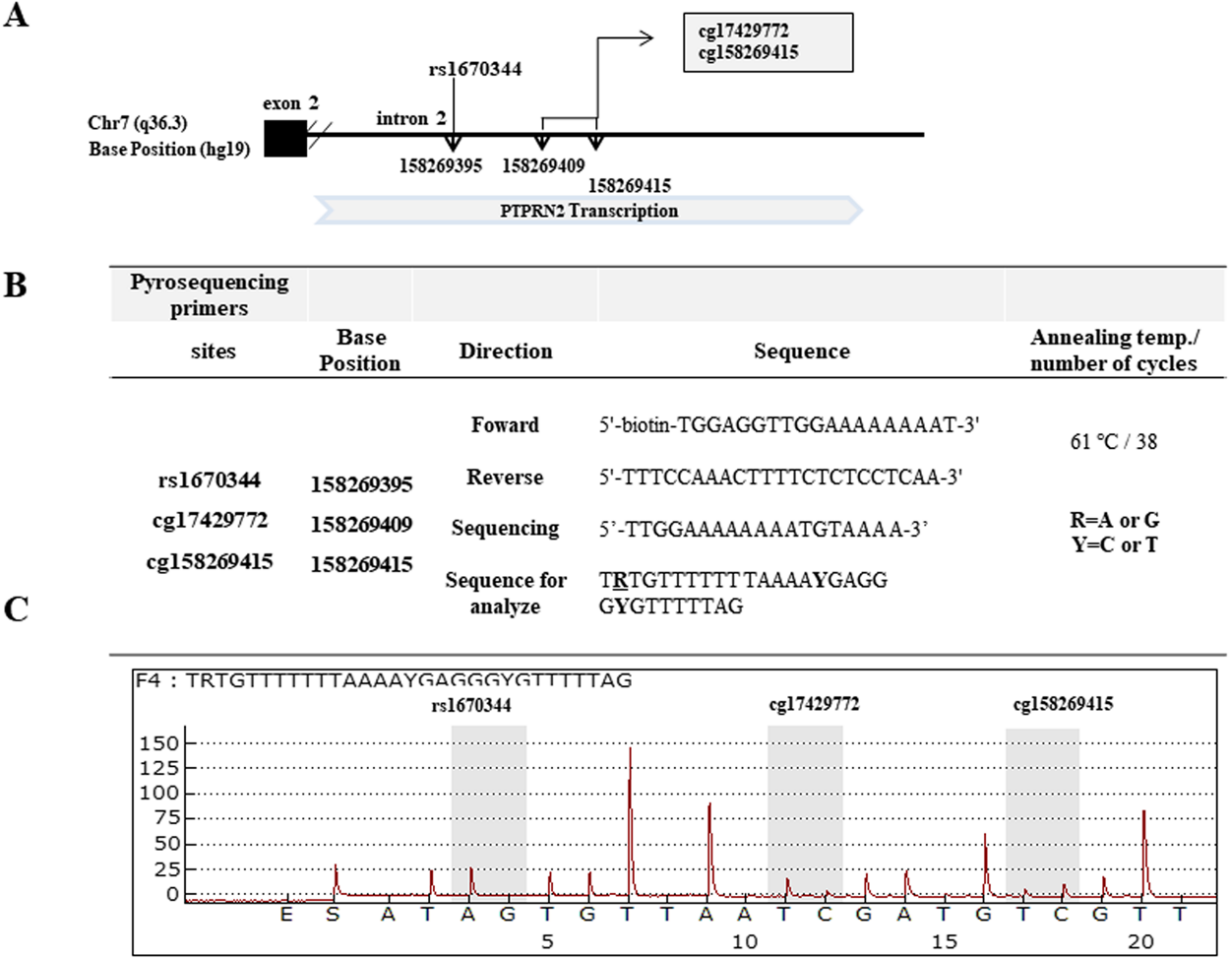
Schematic diagrams of *PTPRN2* locus and pyrosequencing. (**A**) Schematic diagrams of *PTPRN2* locus with rs1670344 and two CpG sites (cg17429772 and cg158269415) in intron 2. The biological position of each site is indicated by arrows(hg19). (**B**) Primer sequences and PCR conditions for pyrosequencing. (**C**) Pyrogram for the experiment.

Polymerase chain reactions (PCRs) of *PTPRN2* were performed for the purpose of pyrosequencing^16^. PCR cycling conditions and sequences of all primers designed with MethPrimer (Biotage AB, Uppsala, Sweden) for cg17429172 and cg158269415 are shown in Fig. 1. Pyrosequencing was conducted using bisulfite treated DNA (Zymo Research, CA). Technical controls for pyrosequencing revealed median methylations of 0% ~ 10% (unmethylated control), and >90% (methylated control), thereby defining detection limits of EpiTect PCR control DNA set (QIAGEN, USA). Pyrosequencing assays were designed, optimized, and performed on a PSQ HS 96A Pyrosequencing System (Pyrosequencing, Westborough, MA, USA) according to the manufacturer’s specifications.

With one assay, quantitation of cg17429772 and cg158269415 was performed with rs1670344 genotyping. Genomic position and gene positions of these two target CpG sites and rs1670344 were analyzed via pyrosequencing. Results are described in Fig. 1. These two CpG methylation sites (cg17429772 and cg158269415) are located downstream from rs1670344 (cg17429772: 14 bp and cg158269415: 20 bp). Finally, we measured DNA methylations in 638 samples (controls, n = 386; cases, n = 252). Pyrosequencing with genotyping was performed using bisulfite-treated DNA. We determined genotyping and CpG methylations at the same time.

### Statistical analyses

Independent sample t-tests were used to compare physical and metabolic characteristics between control and obese case groups. Data are presented as mean ± standard deviation of the mean for ease of interpretation. Multiple regression analyses were performed after adjusting for age and sex.

Statistical analysis for DNA methylation was performed by non-parametric methods because of its bimodal data distribution. Wilcoxon signed-rank test, Kruskal–Wallis test, and Spearman’s correlation test were adopted. Age and sex were used as covariates for the study performed with regression analysis. We used R scripts for all other analytical and graphic processing. Data were visualized using ggplot2 (http://www.r-project.org/).

## Results

General characteristics of obese children with age- and sex-matched controls are summarized in Table 1. We categorized children (n = 252) with an average body mass index (BMI) of 32.69 ± 3.60 as obese and those (n = 386) with an average BMI of 20.23 ± 2.58 as lean controls. There was no significant difference in age (*P* > 0.05) between the two groups. Table 1 also shows averages of BMI, waist-hip-ratio (WHR), fasting glucose levels (FPG), hepatotoxicity measurements, AST (aspartate transaminase), and ALT (alanine aminotransferase), lipid, TC (total cholesterol), TG (triglyceride), HDL (high-density lipoprotein-cholesterol), and LDL (low-density lipoproteins-cholesterol) levels, including significant differences between obese children and controls.

### CpG methylation of childhood obesity

Two CpG methylation sites, cg17429772 and cg158269415, are located at intron 2 of *PTPRN2*. We examined these two CpG sites by pyrosequencing using bisulfite-treated DNA. cg17429772 is located six bases after cg158269415. It was named by chromosome base pair location (hg19). Table 2 shows average distributions of these two CpG methylations in all subjects with childhood obesity (n = 638). Only cg158269415 was particularly differentially methylated between control and obese groups. The methylation was 2.93% higher in cases. It was significantly associated with childhood obesity (*P* = 0.004).

**Table 2:** CpG methylations of PTPRN2 gene in children with childhood obesity.

CpG sites	Position	methylation % of Controls (n = 386)	methylation % of Cases (n = 252)	*P*
cg17429772	chr7:158269409	54.19 ± 11.5	54.05 ± 11.22	0.85
cg158269415	chr7:158269415	21.13 ± 15.21	24.06 ± 14.17	**0**.**004**

Methylation percentage values are indicated by mean ± SD, P values < 0.05 are described in bold. Position: base positions.

To investigate the potential biological significance of cg158269415 methylation, we interrogated CpG methylation with obesity related traits (n = 638). Results of linear regression analysis with beta and *P* values of the methylation of cg158269415 with nine obesity related traits (BMI, WHR, FPG, AST, ALT, TC, TG, HDL, and LDL) are listed by totals, controls, and cases (Table 3). Age and sex are used as covariates for regression analysis.

**Table 3:** Linear regression analysis of cg158269415 methylation with obesity related traits.

Traits		Total subjects (n = 638)	Control subjects (n = 386)	Case subjects (n = 252)
BMI (kg/m^2^)	***beta***	0.04452	−0.016610	0.05306
	***P***	**0**.**0355**	**0**.**04839**	**0**.**0002972**
WHR	***beta***	0.0003438	−0.0003633	0.0005827
	***P***	**1**.**924e-08**	<**2**.**2e-16**	**2**.**237e-08**
FPG (mg/dL)	***beta***	−0.003988	0.02541	−0.07162
	***P***	**2.555e-05**	**9.788e-06**	0.06202
AST (IU/L)	***beta***	0.07789	−0.00276	0.17742
	***P***	**4**.**274e-08**	**1**.**322e-05**	**6**.**276e-05**
ALT (IU/L)	***beta***	0.17692	−0.04406	0.4342
	***P***	**1**.**113e-06**	**0**.**02282**	**5**.**324e-06**
TC (mg/dL)	***beta***	0.07938	−0.01786	0.1567
	***P***	**0**.**000319**	**2**.**95e-07**	0.2636
TG (mg/dL)	***beta***	0.1800	−0.1078	0.2467
	***P***	**0**.**048**	**0**.**03833**	0.1714
HDL (mg/dL)	***beta***	−0.006262	0.05648	−0.02699
	***P***	**0**.**01464**	**0**.**009061**	0.4422
LDL (mg/dL)	***beta***	0.04963	−0.05277	0.1344
	***P***	**0**.**006204**	**4**.**32e-06**	0.08108

BMI (kg/m^2^): Body Mass Index; FPG (mg/dL): Fasting Plasma Glucose; WHR: Waist to Hip Ratio; AST(IU/L): Aspartate transaminase; ALT(IU/L): alanine aminotransferase; TC (mg/dL): Total Cholesterol; TG (mg/dL): Triglyceride; HDL (mg/dL): High-density lipoproteins-Cholesterol; LDL(mg/dL): Low-density lipoproteins-Cholesterol. Raw P values < **0.05** are described in bold characters. Age and sex are used as covariates.

The cg158269415 methylation was significantly correlated with all nine obesity-related traits in total and control groups (*P* < 0.05), but only significant with BMI, WHR, AST, and ALT in the case group (*P* < 0.05). These correlations were positive for seven of nine traits, excluding FPG and HDL. For every 1% increase in cg158269415 in blood cells, BMI increased by 0.04 kg/m^2^ in total subjects, −0.02 kg/m^2^ in the control group, and 0.05 kg/m^2^ in the case group. WHR increased by 0.0003 in the total subjects, −0.0003 in the control group, and 0.0005 in the case group for every 1% increase in cg158269415.

**Figure 2:**
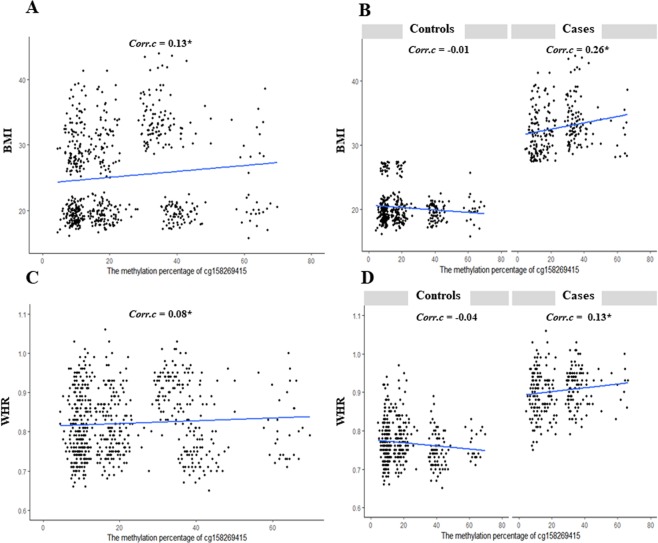
Correlations of cg158269415 with (**A**) BMI in total subjects, (**B**) BMI in controls and obese cases, (**C**) WHR in total subjects, and (**D**) WHR in controls and obese cases. The correlation line is graphed. Spearman correlation coefficients (*Corr*.*c*) are indicated at the top right with statistical significance. *P* < 0.05 is marked with a star (*).

We graphed correlations of cg158269415 with BMI (Fig. 2AB) and WHR (Fig. 2CD) for the total subjects (Fig. 2AC) and control-case groups (Fig. 2BD). cg158269415 methylation was significantly correlated with BMI (Spearman test, *Corr*.*c* = 0.13*) and WHR (Spearman test, *Corr*.*c* = 0.08*) in total subjects. In control and case groups, correlation directions for BMI and WHR were different: negative in controls but positive in case subjects. Correlation directions of cg158269415 with BMI and WHR were significantly different depending on the group. Correlations were insignificantly negative in the control group, but significantly positive in the case group (Spearman test).

### Relation of rs1670344 genotype with cg158269415 methylation

Mechanistic studies for the association of cg158269415 methylation with obesity related traits were performed by investigating nearby rs1670344 genotype which might be important for epigenetic regulation. We conducted pyrosequencing with genotyping of rs1670344 at the same time of CpG methylation measurement. We found 54.4% of GG, 40.4% of AG, and 5.2% of AA genotypes in the total of 638 subjects. By analyzing 386 controls and 252 obese cases, rs1670344 was not associated with childhood obesity, with an odd ratio of 0.98 (raw *P* values > 0.05). By linear regression analysis with age and sex as covariates, rs1670344 had significant associations with WHR (*beta* = −0.0001, *P* = 5.73e-08), FPG (*beta* = −0.07, *P* = 2.52e-05), AST (*beta* = 1.11, *P* = 3.068e-07), ALT (*beta* = 2.13, *P* = 1.486e-05), TC (*beta* = 3.09, *P* = 1.518e-04), HDL (*beta* = 0.6826, *P* = 9.597e-03), and LDL (*beta* = 2.328, *P* = 3.252e-03). However, it had no significant association with BMI (*beta* = 0.09, *P* > 0.05) or TG (*beta* = 0.42, *P* > 0.05). Correlations were positive for seven of the nine traits, excluding WHR and FPG. Logistic and linear regression analyses showed no significant association of rs1670344 with BMI.

**Figure 3:**
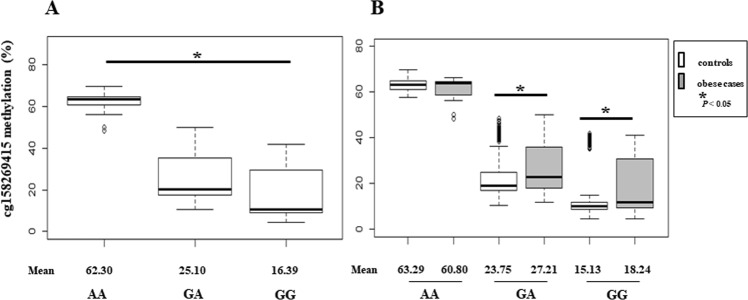
Boxplots of cg158269415 methylation of childhood obesity (n = 638). (**A**) cg158269415 methylations by rs1670344 genotype. AA: homozygous minor genotype; GA: heterozygous genotype; GG: homozygous major genotype. (**B**) cg158269415 methylations between controls and obese cases by rs1670344 genotype. Gray boxes indicate obese cases. *P* values < 0.05 are marked with a star (*).

To investigate genetic and epigenetic interactions in *PTPRN2* gene, we analyzed rs1670344 and cg158269415 genotypes in total, control, and case subjects. Figure 3 shows boxplots of relationships of cg158269415 methylations with genotype. The relationship of CpG methylation with rs1670344 genotypes (GG: homozygous for the major allele, GA: heterozygous, AA: homozygous for the minor allele) is graphed in Fig. 3A. cg158269415 methylation had trimodal distribution patterns that depended on the genotype. The cg158269415 methylation was low at GG, middle at GA, and high at AA (Kruskal–Wallis test, *P* < 0.05). Changes of cg158269415 methylation between AA and GG genotypes were 45.91% on average. Mean values of cg158269415 methylations are indicated under the graph.

cg158269415 methylations in controls and obese cases are graphed by each genotype (Fig. 3B). The degree of CpG methylation of obese cases was significantly higher than that of controls by an average of 3.46% at GA (Wilcoxon test, *P* < 0.05), 3.11% at GG (Wilcoxon test, *P* < 0.05), and −2.49% at AA genotype (Wilcoxon test, *P* > 0.05) (Fig. 3B). Finally, cg158269415 methylations in whole blood cells were strongly associated with childhood obesity under certain genetic background of rs1670344.

**Figure 4:**
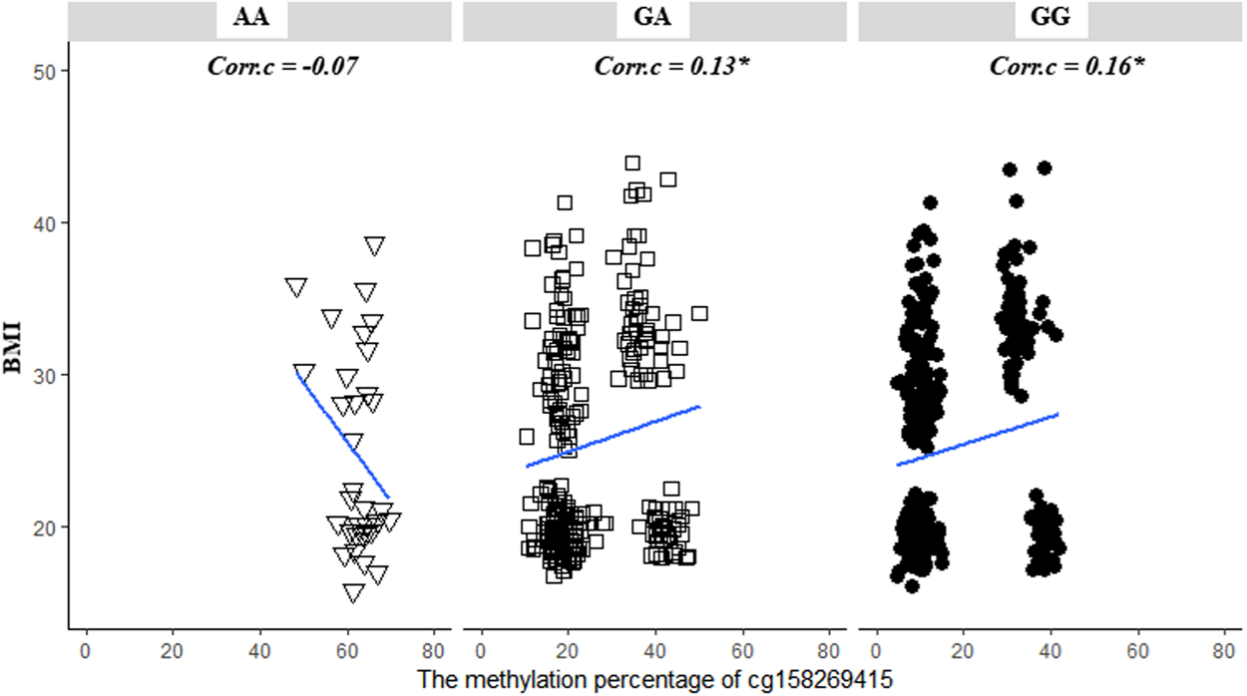
Correlations of cg158269415 methylation with BMI by rs1670344 genotype. AA: homozygous minor genotype, GA: heterozygous genotype, GG: homozygous major genotype. Correlation lines fitted on graphs with spearman correlation coefficients (*Corr*.*c*) indicated in the top of each graph. Statistic significances with *P* value < 0.05 are marked with a star (*).

Our data showed that the correlation of cg158269415 with BMI was different depending on subjects’ rs1670344 genotype. Therefore, we graphed cg158259415 methylations with BMI based on each genotype (Fig. 4). Correlation coefficients for each genotype (AA, AG, GG) are described at the top. Statistic significances with *P* < 0.05 are marked with a star (Spearman test). cg158269415 had a bimodal distribution pattern in GA and GG groups, but not in AA group. Correlation directions and strengths differed depending on genotypes. In AA genotype, cg158269415 showed negative correlation with BMI (Spearman test, *Corr*.*c* = −0.07). However, the correlation was positive in major G allele carriers (Spearman test, *Corr*.*c* = 0.13* in GA and 0.16* in GG).

## Discussion

Results of this study showed that cg158269415 was significantly hypermethylated in the blood of obese children. This study is the first to validate the association of cg158269415 methylation with childhood obesity. Interestingly, the significance and strength of correlations with BMI were different between control and case groups. The correlation of cg158269415 methylation with BMI was strong and significant in case group, suggesting that cg158269415 hypermethylation of obese children might not be directly functional or causal. It might be epigenetic mark left by another process. In addition to BMI and WHR, cg158269415 methylation had positive correlations with five biochemical traits (AST, ALT, TC, TG, and LDL), but negative correlations with two traits (FPG and HDL). DNA methylation associations with BMI may be driven by co-associations with other factors such as hyperlipidemia (TC, TG and LDL).

Previous BMI-related CpG methylation studies in blood showed that increased methylation at the CpG sites located at the HIF3A locus is associated with increases or changes in BMI^15,17,18^. The average of the difference in BMI-related CpG methylation between lean and obese children was found to be less than 3% by pyrosequencing or by Sequenom’s MassARRAY. cg158269415 of *PTPRN2* was hypermethylated by an average of 2.93% in cases. Whether this small changes in DNA methylation from whole blood are causally or consequently related to childhood obesity outcome remains to be determined.

The rs1670344 is located in intron 2. Its functional consequences are currently unclear. The genetic variant, rs1670344, had close effects on nearby obesity-associated cg158269415. This likely reflects the regulation of PTPRN2 gene by epigenetic process. rs1670344 was significantly associated with obesity-related traits and cg158259415 methylation, but not directly with BMI. Our case-control and regression study showed no genetic association of rs1670344 with BMI. rs1670344 genotype might have triggered epigenetic changes of closely located cg158269415 or other CpG sites that had a dynamic role in gaining weight. Environmental factors also may drive epigenetic perturbation of *PTPRN2* based on the rs1670344 genetic background.

The correlation of cg158259415 methylation with BMI was different according to each genotype of rs1670344. It was especially significant and strong in major G allele carriers (GG and GA group). High and positive correlation of CpG methylation with BMI might have come from G allele specific which had bimodal methylation patterns. In AA group, the average of cg158269415 methylation was 62.45% whereas it was 16.39% in GG group. Hypermethylation of cg158269415 in AA group might have a small room for additional DNA methylation by environmental factors.

We investigated differential cg158269415 methylation in blood for obesity. However, the effect of epigenetic perturbation of PTPRN2 remains to be determined depending on cell type. Obesity is an important component of the pathophysiology of immune diseases. Obesity specific cg158269415 in peripheral blood may come from obesity derived from different immune-related functions or immune cell composition in blood between lean and obese children. Epigenome-wide analysis of obesity in peripheral blood cells has shown that dysregulated DNA methylation is associated with inflammation and immune disease^12,19,20^. Especially, the differential methylation of PTPRN2 in naive CD4+ T cell was associated with autoimmune disease, which suggested its role for immune response^12^.

cg158269415 methylation was also significantly associated with FPG. The correlation of cg158269415 methylation with FPG was significant and negative (*beta* = −0.004, *P* = 2.55e-05). rs1670344 also had significant correlation with FPG (*beta* = −0.07, *P* = 2.52e-05). *PTPRN2* as a major autoantigen in insulin-dependent diabetes mellitus may explain its genetic and epigenetic associations with FPG. *PTPRN2* shares >90% sequence identities with rat phogrin and mouse Islet antigen 2 beta (IA-2 beta)^21^. IA-2 beta (−/−) knockout mice show mild glucose intolerance and impaired glucose-stimulated insulin secretion^22^.

In general, a genetic context of the PTPRN2 locus might be important for epigenetic modification of cg158269415 in high-BMI children. Further analysis of these newly-identified genetic and epigenetic factors may be necessary to explain where and how these sophisticated DNA methylation changes come from.
